# Is Myasthenia Gravis a Real Complication of the COVID-19 Vaccine? A Case Report-Based Systematic Review

**DOI:** 10.1155/2022/5009450

**Published:** 2022-09-17

**Authors:** Omid Mirmosayyeb, Elham Moases Ghaffary, Mahsa Mazdak, Zahra Bagheri, Sara Bagherieh, Vahid Shaygannejad

**Affiliations:** ^1^Department of Neurology, School of Medicine, Isfahan University of Medical Sciences, Isfahan, Iran; ^2^Isfahan Neurosciences Research Center, Isfahan University of Medical Sciences, Isfahan, Iran

## Abstract

**Background:**

Myasthenia gravis (MG) is a neuromuscular, autoimmune disease that causes weakness by impairing neuromuscular transmission. According to reports, vaccines can lead to autoimmunity in different ways, and COVID-19 vaccines are suggested to trigger MG. We conducted this systematic review to assess MG patients after the COVID-19 vaccination.

**Methods:**

We collected 231 studies from four databases from inception to 26 March 2022.

**Results:**

4 case studies were selected from 231 research studies, and data were extracted based on inclusion criteria. In all cases, MG was reported following COVID-19 vaccination. Symptoms such as muscle weakness, numbness, and ptosis were common. The MG was confirmed through RNST, MRC, NCS, and AchR-binding antibody titer tests.

**Conclusion:**

Although all cases of MG were diagnosed following appropriate tests, the sample size was small; therefore, further investigation is required to demonstrate the possible association between MG and COVID-19 vaccination.

## 1. Introduction

Myasthenia gravis (MG) is an autoimmune disease caused by antibodies that bind to neuromuscular junction (NMJ) components, disrupting normal function and reducing neuromuscular acetylcholine (ACh) transmission [1–3]. MG, which is characterized by the fatiguability of skeletal muscles and weakness of ocular, bulbar, respiratory, and axial muscles, typically affects ocular muscles at the beginning, resulting in diplopia and intermittent ptosis [4–6]. MG disease is associated with abnormal thymus, defective immune regulation, inflammation, and chronic cell activation [7]. Acetylcholinesterase inhibitors, immunosuppressant agents, steroids, and thymectomies are usually used to treat MG [3].

Viruses from the Coronaviridae family (severe acute respiratory syndrome coronavirus (SARS-CoV)) and Middle East respiratory syndrome coronavirus (MERS-CoV) are human respiratory pathogens. As a result of a new strain of SARS-CoV named SARS-COV-2, a global pandemic ensued in 2019. The patients suffered from mild symptoms such as fatigue, fever, and dry cough. Severe cases can experience acute respiratory distress syndrome (ARDS), cardiac and renal failure, and eventually death [8]. Several instances of COVID-19 infection have resulted in some complications in patients.

Due to the spread of SARS-CoV-2 among countries, global communities have responded to the urgent need for safe and effective COVID-19 vaccines with unprecedented speed and action [9]. There have been several SARS-CoV-2 vaccines developed and available around the world, including mRNA (i.e., Pfizer-BioNTech [BNT162b2] and Moderna [mRNA-1273]), viral vector (i.e., Johnson and Johnson's Janssen [Ad26.COV2-S] and Oxford-AstraZeneca [ChAdOx1 nCoV-19]), and inactivated vaccines (Covaxin, CoronaVac, and Sinopharm), since December 2020. These vaccines have shown high efficacy and safety in protecting against SARS-CoV-2 infection [10, 11].

The common adverse events of the vaccine were redness, swelling, body pain, fatigue, headache, and fever [12, 13]. COVID-19 vaccine can rarely affect the central nervous system (CNS) and peripheral nervous system (PNS) and has been associated with neurological manifestations including stroke, Guillain-Barré syndrome (GBS), Bell's palsy, autoimmune diseases (AID), cerebral venous sinus thrombosis (CVST), transverse myelitis (TM), acute disseminated encephalomyelitis (ADEM), myalgia and arthralgia, and acute demyelinating polyneuropathy [14–16].

Although COVID-19 vaccines generate broad immunity against the infection and are the best and safest method for controlling the pandemic, a few cases and reports have shown onset MG associated with the COVID-19 vaccine. It is crucial to recognize less common symptoms related to COVID-19 vaccine-associated MG, including dysphagia and dysarthria and pay attention to the timing of vaccination. MG can be worsened and triggered by infection; however, no specific association with viruses or pathogens has been demonstrated. The underlying pathogenesis of MG is unclear but it is speculated that immune response changes following vaccination could generate antibodies against AChRs [14, 17–19].

The purpose of this systematic review was to collect all published cases of MG after receiving the COVID-19 vaccine.

## 2. Methods

### 2.1. Literature Study

At first, we systematically searched five databases, including Pubmed (Medline), Embase, Scopus, Web of Science, and Google Scholar, with the MeSH terms including “myasthenia gravis” and “COVID-19 Vaccines” (see the supplementary file) for identifying all studies from inception to 26 March 2022. In addition, all syntaxes were customized for each database.

### 2.2. Inclusion and Exclusion Criteria

All case report/series studies which included MG cases following the COVID-19 vaccine were included in the inclusion criteria. The exclusion criteria were as follows: articles written in any language other than English, review articles, animal studies, hypotheses, In vitro studies, as well as patients with MG who got vaccines and whose symptoms became worse after vaccination.

### 2.3. Study Selection

First, two researchers (ZB and MM) reviewed the related articles separately and selected desired studies afterward. Any differences in view between included and excluded studies were resolved by a senior reviewer (SV) comment.

### 2.4. Data Extraction

Two authors (ZB and MM) performed the extraction of data independently according to qualified information, including demographic data, comorbidities, name of the vaccine, time interval/dose, vaccine side effects, MG first signs and symptoms, physical examination findings, laboratory finding, electromyography (EMG) findings, radiologic findings, acute treatment, main treatment, and outcome(Table 1).

### 2.5. Quality Assessment

A systematic review evaluation tool, Joanna Brigs Institute (JBI), was used to assess the quality and risk of bias of each study [20]. Two researchers (MM and ZB) evaluated all studies, and the senior researcher (OM) resolved any disagreement. 4 options for evaluation were available “Yes,” “No,” “Unclear,” and “Not applicable.” Moreover, “Yes” responses were summarized from 0 to 8. Articles with a score lower than 4 are considered low quality, and those with a score higher than 4 are considered high quality (Tables 2 and 3).

## 3. Result

**Figure 1:**
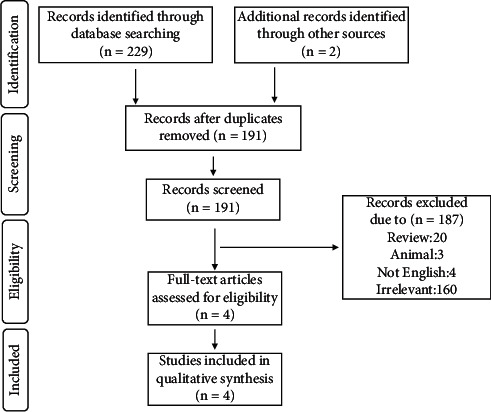
PRISMA flow diagram: includes details our search and selection process applied during the systematic review.

Using the PRISMA flowchart (Figure 1), studies were selected based on exclusion and inclusion criteria. At first, 229 articles were identified from 4 databases, then 38 records were removed before screening based on their duplication. 191 records were screened, and 187 were excluded based on their irrelevancy. Full-text articles were collected, and 4 articles were used in our study. Table 1 provides demographic information for 5 patients. 4 (75%) patients were male, the mean (SD) of their age was 66.6 (19.2), most of them got the BNT162b2 vaccine, and the main treatments of patients were pyridostigmine and prednisone. Finally, available data demonstrated that one patient was intubated while 4 recovered.

## 4. Discussion

Vaccines have profoundly affected human health and longevity, with statistics showing around nine million lives being saved each year by vaccination. Smallpox has been eradicated from the planet thanks to vaccination [21]. Vaccines are intended to make antibodies against pathogens, or trigger the immune system to deal with them more effectively. Even though modern vaccines are made on different platforms (whole germ, viral vector, nucleic acid-based, subunit, and nanoparticle-based vaccines), they all have components associated with some neurological damage and autoimmune side effects [22, 23]. We systematically review the existing publications on cases of MG following SARS-CoV-2 vaccination.

### 4.1. SARS-CoV-2 Vaccines

The COVID-19 pandemic has affected human lives in various social, economic, and health aspects. These issues include lockdowns, economic slowdowns, limited freedoms, being infected, and losing loved ones [24]. Vaccination seems to be the most efficacious intervention in combating this pandemic [25]. At the time of writing, there are 149 SARS-COV-2 vaccines in clinical development and 195 vaccines in preclinical development, according to the WHO database [26]. In December 2020, the first-ever vaccine for combating SARS-COV-2 infection was approved as an mRNA-based vaccine, BNT162b2 [27]. Up to now, available vaccines include mRNA vaccines (CVnCoV, mRNA-1273, and BNT16b2), inactivated vaccines (Wuhan Sinopharm, CoronaVac, NVX-COV2373, BBIBP-CorV, Covaxin, KoviVac, QazVac, and COVIran Barekat), viral vector vaccines (Sputnik V Light, Sputnik V, AZD1222, Ad26.COV2.S, and Ad5-nCoV), and protein-based vaccines (Abdala, ZF200, and EpiVacCorona) [11].

### 4.2. SARS-CoV-2 Vaccine-Induced MG

Even though COVID-19 vaccines were administered to protect against SARS-COV-2 infection not long ago, a variety of adverse effects have been reported. Molecular mimicry between SARS-CoV-2 molecules and human antigens can cause AID in vaccine receivers [28]. Vojdani and Kharrazian proved the cross-reactivity of 21 human tissue antigens with the SARS-CoV-2 antibodies, which can be responsible for the fact that COVID-19 infections and SARS-COV-2 mRNA vaccines trigger autoimmunity against gastrointestinal, cardiovascular, nervous systems, and connective tissues [29]. It may also result in aberrant activation of acquired and innate immunity when mRNA vaccines trigger a cascade of immune reactions [30]. In addition, certain adjuvants are likely to cause self auto-reactive T cells differentiation, which will damage the host tissues [31]. The adjuvants are molecules that induce innate immunity by activating the pattern recognition receptors (PRRs). Therefore, vaccines commonly contain them to grow immunity against antigens [32, 33]. SARS-CoV-2 adjuvanticity of vaccines works as toll-like receptors (TLR)-7/8, or TLR-9 agonist and is novel compared to previous vaccines. This can be a new pathogenic mechanism causing immune-mediated diseases in people [34, 35].

The findings of multiple studies suggest HPV vaccinations may cause MG either as an adverse event or incidentally without a relationship between them [36]. It has been hypothesized that an antibody that cross-reacts with autonomic ganglia, neurons, and cardiovascular proteins could be synthesized by the HPV vaccine epitope and that the production of the antibody could activate cytotoxic T cells by binding to acetylcholine receptors [36, 37]. The other number of factors, including hepatitis B virus (HBV) [38, 39], bacillus Calmette–Guerin (BCG) [40], and the influenza vaccines [41], led to MG.

Chavez and Pougnier reported an 82-year-old man with slurred speech symptoms two days after receiving his second dose of an mRNA-based COVID-19 vaccine. Due to the high titers of AchR-antibodies in his serum and his EMG test results, he was confirmed to develop MG following vaccination. Also, after two weeks of treatment, he was bothered by droopy eyelids [18]. Based on the very short time between vaccine injection and showing symptoms, bystander immunity can be the underlying explanation, in which a continuous immune response and inflammation allow autoantigens to be exposed and autoreactive T lymphocytes to be activated [42]. In contrast, Lee et al. reported a 33-year-old female with myalgia, generalized weakness, diplopia, and ptosis following the second dose of an mRNA-based COVID-19 vaccine, who developed thymic hyperplasia, as well as an absence of antibodies to AchR and muscle-specific tyrosine kinase (MuSK). Nevertheless, it is still hypothesized that alternations in immune response following vaccination may produce antibodies against AchR and this seronegative MG patient may have antibodies that cannot be detected by current assay methods [14].

Interestingly, Tagliaferri et al. reported an MG crisis following an mRNA-based vaccine in a patient diagnosed with MG 5 years prior. They acknowledged that the cytokine storm caused an MG flare in their patient, especially when he showed improvement after low-dose steroid therapy [3], which decreased lymphocyte proliferation, differentiation, and cytokine expression [43]. Moreover, an MG crisis caused the death of an 86-year-old patient after the vaccination [44]. Despite all these, the capacity of the SARS-COV-2 virus to cause neurological damage is way much more than COVID-19 vaccines [45–51].

As a strong explanation, it can be said that inflammatory responses in MG are stimulated and sustained by TLR signaling pathways activation [52–57]. On the other hand, activating adaptive immunity in response to vaccines is also controlled by TLRs pathways. These findings show that TLRs are involved in vaccine effectiveness and MG pathogenesis [58].

Moreover, a TLR3 agonist known as polyinosine-polycytidylic acid can cause changes in the thymus (which is supposed to be related to MG pathogenesis [59]) and flares MG symptoms through imitating virus double-stranded RNA (dsRNA), which is the replicative virus component [55]. Therefore, any COVID-19 vaccine that contains adjuvant or pathogen antigen molecule mimicking dsRNA can activate the TLR3 pathway and perhaps cause an autoimmune response against acetylcholine receptors [58].

We systematically evaluated all cases presenting MG patients following COVID-19 with different vaccines injection. Our systematic review is the first conducted in this field. We were constrained by some limitations, such as the small number of cases indicating that they are not representative of the population. More studies are needed to clarify the actual relationship between COVID-19 vaccines and MG in healthy individuals or MG flare in those who are susceptible.

## 5. Conclusion

While COVID-19 vaccines provide broad immunity against the infection and are among the most effective and safe methods of controlling this pandemic, the onset of MG has been associated with the vaccine in a small number of cases; nonetheless, given the small number of cases reported, it cannot be conceded that MG is necessarily a complication of the COVID-19 different vaccines, and more data and cases are needed for the conclusion.

## Figures and Tables

**Table 1:** Patients' characteristics in myasthenia gravis diagnosis following COVID-19 vaccines.

Author/ Year/country	Age/gender	Comorbidities	Name of vaccine	Time interval/dose	Vaccine side effects	MG first signs and symptoms	Physical examination findings	Laboratory findings	EMG findings	Radiologic findings	Acute treatment	Main treatment	Final outcome
Augustine Chavez et al./August 2021/USA [18]	82/M	Laryngeal cancer (hemi-laryngectomy) and barrett's esophagus, stage 3 CKD	BNT162b2	2 days/2nd	NR	Slurred speech, perioral numbness, and difficulty in chewing and spitting	Hoarse voice, normal neurologic examination (healthy cranial nerve, no cognitive, sensory, or motor deficits)	AchR-binding antibody titer: 11.4, AchR modulating antibody: 93%, and Striational antibody titer: 1 : 245760	RNST: decrement response	Head CT scan: changes in white matter (as a result of aging), and no acute intracranial abnormality, MRI: no laryngeal tumor	NR	Pyridostigmin, speech therapy, steroids, and IVIG	Discharged (transferred to a rehabilitation center)
Myung Ah Lee et al./November 2021/Korea [14]	33/F	No medical history	BNT162b2	The evening of the injection/ 2nd	Myalgia	Generalized weakness, binocular diplopia, bilateral ptosis, difficulty with moving her arms and neck, dysarthria, and dysphagia	Both lower and upper extremities MRC scale: 4.5, sensory and motor NCS results: normal	AchR antibody titer: <0.02 nmol/L and Neostigmine test confirmed the diagnosis of MG	RNST: remarkable decrement response of right orbicularis oculi	Chest CT: mild thymus hyperplasia	NR	Oral pyridostigmine (360 mg/day)	Recovered and discharged

Abdulla Watad et al./March 2021/Israel [17]	72/M	Recurrent pericarditis (colchicine-treated)	BNT162b2	1 day/2nd	NR	NR	NR	NR	Decrement response on the shoulder and facial muscles (28 to 46%)	NR	PLEX	Prednisone 60 mg	Recovered
	73/M	NR	BNT162b2	7 days/2nd	NR	Started with ocular signs continued with respiratory symptoms and bulbar signs	NR	NR	Borderline decrement but remarkable pathologic jitter	NR	PLEX	Pyridostigmin, prednisone	Intubated
Giuliana Galassi et al./January 2022/Italy [19]	73/M	Mild hypertension and myocardial infarction (smoker)	ChAdOx1	8 days/1st	Myalgia and Fever (up to 39°C)	Psoriasis (in both elbows) and Painless left-sided ptosis	NR	RF: 240 IU/ml, COVID-19 PCR: Negative, Anti- AChR antibody titer: 1.9 nmol/l	RNST: Decrement response in nasalis muscle (14.7%) and Normal response in the ulnar and accessory nerves	Brain CT: normal Chest CT: No thymoma	Paracetamol	Pyridostigmine bromide (240 mg per day)	Recovered

M: male, F: female, CKD: chronic kidney disease, Plex: plasma exchange, AchR: acetylcholine receptor, RNST: repetitive nerve stimulation test, CT: computed tomography, MRC: muscle power assessment, NCS: nerve conduction studies, Musk: muscle-specific kinase, RF: rheumatoid factor, PCR: polymerase chain reaction, and NR: not reported.

**Table 2:** Quality assessment based on the JBI tool for case reports.

	Giuliana Galassi et al.	Myung Ah Lee et al.	Augustine Chavez et al.
1. Were the patient's demographic characteristics clearly described?	Yes	Yes	Yes
2. Was the patient's history clearly described and presented as a timeline?	Yes	Not clear	Yes
3. Was the current clinical condition of the patient on presentation clearly described?	Yes	Yes	Yes
4.Were diagnostic tests or assessment methods and the results clearly described?	Yes	Yes	Yes
5. Was the intervention (s) or treatment procedure (s) clearly described?	Yes	Yes	Not clear
6. Was the postintervention clinical condition clearly described?	Yes	Yes	Yes
7. Were adverse events (harms) or unanticipated events identified and described?	Yes	Yes	Yes
8. Does the case report provide takeaway lessons?	Yes	Yes	Yes

**Table 3:** Quality assessment based on the JBI tool for case series.

	Abdulla Watada et al
1. Were there clear criteria for inclusion in the case series?	Yes
2. Was the condition measured in a standard, reliable way for all participants included in the case series?	Yes
3. Were valid methods used for the identification of the condition for all participants included in the case series?	Yes
4. Did the case series have consecutive inclusion of participants?	Yes
5. Did the case series have complete inclusion of participants?	Yes
6. Was there clear reporting of the demographics of the participants in the study?	Yes
7. Was there clear reporting of clinical information of the participants?	Not clear
8. Were the outcomes or follow-up results of cases clearly reported?	Yes
9. Was there clear reporting of the presenting site (s)/clinic (s) demographic information?	Yes
10. Was statistical analysis appropriate?	Yes
Overall	9 out of 10

## Data Availability

The datasets analyzed during the current study are available upon request with no restriction.

## Supplementary Materials

Supplementary Materials Table S1. The customized syntaxes based on each database. .

## References

[1] Payet C. A., You A., Fayet O.-M., Dragin N., Berrih-Aknin S., Le Panse R. (2022). Myasthenia gravis: an acquired interferonopathy?. *Cells*.

[2] Meriggioli M. N., Sanders D. B. (2012). Muscle autoantibodies in myasthenia gravis: beyond diagnosis?. *Expert Review of Clinical Immunology*.

[3] Tagliaferri A. R., Narvaneni S., Azzam M. H., Grist W. (2021). A case of COVID-19 vaccine causing a myasthenia gravis crisis. *CUREUS*.

[4] Hehir M. K., Silvestri N. J. (2018). Generalized myasthenia gravis. *Neurologic Clinics*.

[5] Lazaridis K., Tzartos S. J. (2020). Autoantibody specificities in myasthenia gravis; implications for improved diagnostics and therapeutics. *Frontiers in Immunology*.

[6] Silvestri N., Wolfe G. (2012). Myasthenia gravis. *Seminars in Neurology*.

[7] Vilquin J.-T., Bayer A. C., Le Panse R., Berrih-Aknin S. (2019). The muscle is not a passive target in myasthenia gravis. *Frontiers in Neurology*.

[8] Rothan H. A., Byrareddy S. N. (2020). The epidemiology and pathogenesis of coronavirus disease (COVID-19) outbreak. *Journal of Autoimmunity*.

[9] Flanagan K. L., MacIntyre C. R., McIntyre P. B., Nelson M. R. (2021). SARS-CoV-2 vaccines: where are we now?. *The Journal of Allergy and Clinical Immunology: In Practice*.

[10] Briggs F. B. S., Mateen F. J., Schmidt H. (2022). COVID-19 vaccination reactogenicity in persons with multiple sclerosis. *Neurology-Neuroimmunology Neuroinflammation*.

[11] Fiolet T., Kherabi Y., MacDonald C.-J., Ghosn J., Peiffer-Smadja N. (2022). Comparing COVID-19 vaccines for their characteristics, efficacy and effectiveness against SARS-CoV-2 and variants of concern: a narrative review. *Clinical Microbiology and Infection*.

[12] Kaur R. J., Dutta S., Bhardwaj P. (2021). Adverse events reported from COVID-19 vaccine trials: a systematic review. *Indian Journal of Clinical Biochemistry*.

[13] Al Khames Aga Q. A., Alkhaffaf W. H., Hatem T. H. (2021). Safety of COVID-19 vaccines. *Journal of Medical Virology*.

[14] Lee M. A., Lee C., Park J. H., Lee J. H. (2022). Early-onset myasthenia gravis following COVID-19 vaccination. *Journal of Korean Medical Science*.

[15] Kaulen L. D., Doubrovinskaia S., Mooshage C. (2022). Neurological autoimmune diseases following vaccinations against SARS-CoV-2: a case series. *European Journal of Neurology*.

[16] Garg R. K., Paliwal V. K. (2022). Spectrum of neurological complications following COVID-19 vaccination. *Neurological Sciences*.

[17] Watad A., De Marco G., Mahajna H. (2021). Immune-mediated disease flares or new-onset disease in 27 subjects following mrna/dna sars-cov-2 vaccination. *Vaccines*.

[18] Chavez A., Pougnier C. (2021). A case of COVID-19 vaccine associated new diagnosis myasthenia gravis. *Journal of Primary Care and Community Health*.

[19] Galassi G., Rispoli V., Iori E., Ariatti A., Marchioni A. (2022). Coincidental onset of ocular myasthenia gravis following ChAdOx1 n-CoV-19 vaccine against severe acute respiratory syndrome coronavirus 2 (SARS-CoV-2). *The Israel Medical Association Journal: IMAJ*.

[20] Lockwood C., Munn Z., Porritt K., Qualitative research synthesis: methodological guidance for systematic reviewers utilizing meta-aggregation (2015). International Journal of Evidence-Based Healthcare.

[21] Unicef U. N. C. F. (2019). *Vaccines Bring Control., Diseases Under*.

[22] Lu L., Xiong W., Mu J. (2021). The potential neurological effect of the COVID-19 vaccines: a review. *Acta Neurologica Scandinavica*.

[23] Ismail I. I., Salama S. (2022). A systematic review of cases of CNS demyelination following COVID-19 vaccination. *Journal of Neuroimmunology*.

[24] Haug N., Geyrhofer L., Londei A. (2020). Ranking the effectiveness of worldwide COVID-19 government interventions. *Nature Human Behaviour*.

[25] Saxena S., Skirrow H., Bedford H. (2020). Routine vaccination during COVID-19 pandemic response. *BMJ*.

[26] WHO - R&D Blue Print (2022). *COVID-19 Vaccine Tracker and Landscape - 28 January 2022*.

[27] Ooi E. E., Dhar A., Petruschke R., Locht C., Buchy P., Low J. G. H. (2022). Use of analgesics/antipyretics in the management of symptoms associated with COVID-19 vaccination. *Npj Vaccines*.

[28] Kanduc D., Shoenfeld Y. (2020). Molecular mimicry between SARS-CoV-2 spike glycoprotein and mammalian proteomes: implications for the vaccine. *Immunologic Research*.

[29] Vojdani A., Kharrazian D. (2020). Potential antigenic cross-reactivity between SARS-CoV-2 and human tissue with a possible link to an increase in autoimmune diseases. *Clinical Immunology*.

[30] Talotta R. (2021). Do COVID-19 RNA-based vaccines put at risk of immune-mediated diseases? In reply to ‘potential antigenic cross-reactivity between SARS-CoV-2 and human tissue with a possible link to an increase in autoimmune diseases. *Clinical Immunology*.

[31] Goriely S., Goldman M. (2007). From tolerance to autoimmunity: is there a risk in early life vaccination?. *Journal of Comparative Pathology*.

[32] Bragazzi N. L., Watad A., Sharif K. (2017). Advances in our understanding of immunization and vaccines for patients with systemic lupus erythematosus. *Expert Review of Clinical Immunology*.

[33] Toussirot É., Bereau M. (2016). Vaccination and induction of autoimmune diseases. *Inflammation & Allergy-Drug Targets*.

[34] Tatematsu M., Funami K., Seya T., Matsumoto M. (2018). Extracellular RNA sensing by pattern recognition receptors. *Journal of Innate Immunity*.

[35] Teijaro J. R., Farber D. L. (2021). COVID-19 vaccines: modes of immune activation and future challenges. *Nature Reviews Immunology*.

[36] Chung J. Y., Lee S. J., Shin B.-S., Kang H. G. (2018). Myasthenia gravis following human papillomavirus vaccination: a case report. *BMC Neurology*.

[37] Pinto L. A., Castle P. E., Roden R. B. (2005). HPV-16 L1 VLP vaccine elicits a broad-spectrum of cytokine responses in whole blood. *Vaccine*.

[38] Louzir B., Othmani S., Battikh R. (2003). Myasthenia after hepatitis B vaccination. *Therapie*.

[39] Stübgen J.-P. (2010). Neuromuscular disorders associated with Hepatitis B vaccination. *Journal of the Neurological Sciences*.

[40] Takizawa T., Kojima M., Suzuki S. (2017). New onset of myasthenia gravis after intravesical Bacillus Calmette-Guerin: a case report and literature review. *Medicine (Baltimore)*.

[41] Wang F., Xiang T., He L., Wang J. (2021). Laryngeal myasthenia gravis following influenza vaccination: a case report and literature review. *Human Vaccines and Immunotherapeutics*.

[42] Wraith D. C., Goldman M., Lambert P. H. (2003). Vaccination and autoimmune disease: what is the evidence?. *Lancet*.

[43] Kragballe K. (1989). Topical corticosteroids: mechanisms of action. *Acta Dermato-Venereologica, Supplementum*.

[44] Flores M., Kewan T., Mushtaq K. (2021). Characteristics and outcomes of adverse events after COVID-19 vaccination. *Journal of the American College of Emergency Physicians open*.

[45] Ellul M. A., Benjamin L., Singh B. (2020). Neurological associations of COVID-19. *The Lancet Neurology*.

[46] Needham E. J., Chou S. H.-Y., Coles A. J., Menon D. K. (2020). Neurological implications of COVID-19 infections. *Neurocritical Care*.

[47] Filatov A., Sharma P., Hindi F., Espinosa P. S. (2020). Neurological complications of coronavirus disease (COVID-19): encephalopathy. *Cureus*.

[48] Jarrahi A., Ahluwalia M., Khodadadi H. (2020). Neurological consequences of COVID-19: what have we learned and where do we go from here?. *Journal of Neuroinflammation*.

[49] Beghi E., Feigin V., Caso V., Santalucia P., Logroscino G. (2020). COVID-19 infection and neurological complications: present findings and future predictions. *Neuroepidemiology*.

[50] Siow I., Lee K. S., Zhang J. J. Y., Saffari S. E., Ng A., Young B. (2021). Stroke as a neurological complication of COVID-19: a systematic review and meta-analysis of incidence, outcomes and predictors. *Journal of Stroke and Cerebrovascular Diseases*.

[51] Collantes M. E. V., Espiritu A. I., Sy M. C. C., Anlacan V. M. M., Jamora R. D. G. (2021). Neurological manifestations in COVID-19 infection: a systematic review and meta-analysis. *The Canadian Journal of Neurological Sciences*.

[52] Wang Y. Z., Yan M., Tian F. F. (2013). Possible involvement of toll-like receptors in the pathogenesis of myasthenia gravis. *Inflammation*.

[53] Bernasconi P., Barberis M., Baggi F. (2005). Increased Toll-like receptor 4 expression in thymus of myasthenic patients with thymitis and thymic involution. *The American Journal of Pathology*.

[54] Robinet M., Maillard S., Cron M. A., Berrih-Aknin S., Le Panse R. (2017). Review on toll-like receptor activation in myasthenia gravis: application to the development of new experimental models. *Clinical Reviews in Allergy & Immunology*.

[55] Cufi P., Dragin N., Weiss J. M. (2013). Implication of double-stranded RNA signaling in the etiology of autoimmune myasthenia gravis. *Annals of Neurology*.

[56] Cordiglieri C., Marolda R., Franzi S. (2014). Innate immunity in myasthenia gravis thymus: pathogenic effects of Toll-like receptor 4 signaling on autoimmunity. *Journal of Autoimmunity*.

[57] Cavalcante P., Galbardi B., Franzi S. (2016). Increased expression of Toll-like receptors 7 and 9 in myasthenia gravis thymus characterized by active Epstein–Barr virus infection. *Immunobiology*.

[58] Zhou Q., Zhou R., Yang H., Yang H. (2021). To be or not to be vaccinated: that is a question in myasthenia gravis. *Frontiers in Immunology*.

[59] Utsugisawa K., Nagane Y. (2011). [Thymic abnormalities in patients with myasthenia gravis]. *Brain Nerve*.

