# Supplementary material for: Is Myasthenia Gravis a Real Complication of the COVID-19 Vaccine? A Case Report-Based Systematic Review
Source: Can J Infect Dis Med Microbiol. 2022 Sep 17;2022:5009450. doi: 10.1155/2022/5009450 (PMC9509275; doi:10.1155/2022/5009450)
Supplement: Supplementary Materials — Table S1. The customized syntaxes based on each database. . [file 5009450.f1.docx]

| PubMed  **Supplementary Description**  **Table. S1.** The customized syntaxes based on each database |
| --- |
| (“myasthenia gravis” OR (“Myasthenia Gravis” AND Ocular) OR “Ocular Myasthenia Gravis” OR (“Myasthenia Gravis” AND Generalized) OR “Generalized Myasthenia Gravis” OR “Muscle-Specific Receptor Tyrosine Kinase Myasthenia Gravis” OR “Muscle Specific Receptor Tyrosine Kinase Myasthenia Gravis” OR “Muscle-Specific Tyrosine Kinase Antibody Positive Myasthenia Gravis” OR “Muscle Specific Tyrosine Kinase Antibody Positive Myasthenia Gravis” OR “MuSK MG” OR “MuSK Myasthenia Gravis” OR (“Myasthenia Gravis” AND MuSK) OR “Anti-MuSK Myasthenia Gravis” OR “Anti MuSK Myasthenia Gravis” OR (“Myasthenia Gravis” AND Anti-MuSK))  AND  (“COVID 19 Vaccines” OR (Vaccines AND COVID-19) OR “COVID-19 Virus Vaccines” OR “COVID 19 Virus Vaccines” OR (Vaccines AND “COVID-19 Virus”) OR (“Virus Vaccines” AND COVID-19) OR “COVID-19 Virus Vaccine” OR “COVID 19 Virus Vaccine” OR (Vaccine AND “COVID-19 Virus”) OR (“Virus Vaccine” AND COVID-19) OR “COVID19 Virus Vaccines” OR (Vaccines AND “COVID19 Virus”) OR (“Virus Vaccines” AND COVID19) OR “COVID19 Virus Vaccine” OR (Vaccine AND “COVID19 Virus”) OR (“Virus Vaccine” AND COVID19) OR “COVID19 Vaccines” OR (Vaccines AND COVID19) OR “COVID19 Vaccine” OR (Vaccine AND COVID19) OR “SARS-CoV-2 Vaccines” OR “SARS CoV 2 Vaccines” OR (Vaccines AND SARS-CoV-2) OR “SARS-CoV-2 Vaccine” OR “SARS CoV 2 Vaccine” OR (Vaccine AND SARS-CoV-2) OR “SARS2 Vaccines” OR (Vaccines AND SARS2) OR “SARS2 Vaccine” OR (Vaccine AND SARS2) OR “Coronavirus Disease 2019 Vaccines” OR “Coronavirus Disease 2019 Vaccine” OR “Coronavirus Disease 2019 Virus Vaccine” OR “Coronavirus Disease 2019 Virus Vaccines” OR “Coronavirus Disease-19 Vaccines” OR “Coronavirus Disease 19 Vaccines” OR (Vaccines AND “Coronavirus Disease-19”) OR “Coronavirus Disease-19 Vaccine” OR “Coronavirus Disease 19 Vaccine” OR (Vaccine AND “Coronavirus Disease-19”) OR “COVID 19 Vaccine” OR (Vaccine AND “COVID 19”) OR “2019-nCoV Vaccine” OR “2019 nCoV Vaccine” OR (Vaccine AND 2019-nCoV) OR “2019 Novel Coronavirus Vaccines” OR “2019 Novel Coronavirus Vaccine” OR “2019-nCoV Vaccines” OR “2019 nCoV Vaccines” OR (Vaccines AND 2019-nCoV) OR “COVID-19 Vaccine” OR (Vaccine AND COVID-19) OR “SARS Coronavirus 2 Vaccines”) |
| EMBASE |
| (‘myasthenia gravis’ OR (‘Myasthenia Gravis’ AND Ocular) OR ‘Ocular Myasthenia Gravis’ OR (‘Myasthenia Gravis’ AND Generalized) OR ‘Generalized Myasthenia Gravis’ OR ‘Muscle-Specific Receptor Tyrosine Kinase Myasthenia Gravis’ OR ‘Muscle Specific Receptor Tyrosine Kinase Myasthenia Gravis’ OR ‘Muscle-Specific Tyrosine Kinase Antibody Positive Myasthenia Gravis’ OR ‘Muscle Specific Tyrosine Kinase Antibody Positive Myasthenia Gravis’ OR ‘MuSK MG’ OR ‘MuSK Myasthenia Gravis’ OR (‘Myasthenia Gravis’ AND MuSK) OR ‘Anti-MuSK Myasthenia Gravis’ OR ‘Anti MuSK Myasthenia Gravis’ OR (‘Myasthenia Gravis’ AND Anti-MuSK))  AND  (‘COVID 19 Vaccines’ OR (Vaccines AND COVID-19) OR ‘COVID-19 Virus Vaccines’ OR ‘COVID 19 Virus Vaccines’ OR (Vaccines AND ‘COVID-19 Virus’) OR (‘Virus Vaccines’ AND COVID-19) OR ‘COVID-19 Virus Vaccine’ OR ‘COVID 19 Virus Vaccine’ OR (Vaccine AND ‘COVID-19 Virus’) OR (‘Virus Vaccine’ AND COVID-19) OR ‘COVID19 Virus Vaccines’ OR (Vaccines AND ‘COVID19 Virus’) OR (‘Virus Vaccines’ AND COVID19) OR ‘COVID19 Virus Vaccine’ OR (Vaccine AND ‘COVID19 Virus’) OR (‘Virus Vaccine’ AND COVID19) OR ‘COVID19 Vaccines’ OR (Vaccines AND COVID19) OR ‘COVID19 Vaccine’ OR (Vaccine AND COVID19) OR ‘SARS-CoV-2 Vaccines’ OR ‘SARS CoV 2 Vaccines’ OR (Vaccines AND SARS-CoV-2) OR ‘SARS-CoV-2 Vaccine’ OR ‘SARS CoV 2 Vaccine’ OR (Vaccine AND SARS-CoV-2) OR ‘SARS2 Vaccines’ OR (Vaccines AND SARS2) OR ‘SARS2 Vaccine’ OR (Vaccine AND SARS2) OR ‘Coronavirus Disease 2019 Vaccines’ OR ‘Coronavirus Disease 2019 Vaccine’ OR ‘Coronavirus Disease 2019 Virus Vaccine’ OR ‘Coronavirus Disease 2019 Virus Vaccines’ OR ‘Coronavirus Disease-19 Vaccines’ OR ‘Coronavirus Disease 19 Vaccines’ OR (Vaccines AND ‘Coronavirus Disease-19’) OR ‘Coronavirus Disease-19 Vaccine’ OR ‘Coronavirus Disease 19 Vaccine’ OR (Vaccine AND ‘Coronavirus Disease-19’) OR ‘COVID 19 Vaccine’ OR (Vaccine AND ‘COVID 19’) OR ‘2019-nCoV Vaccine’ OR ‘2019 nCoV Vaccine’ OR (Vaccine AND 2019-nCoV) OR ‘2019 Novel Coronavirus Vaccines’ OR ‘2019 Novel Coronavirus Vaccine’ OR ‘2019-nCoV Vaccines’ OR ‘2019 nCoV Vaccines’ OR (Vaccines AND 2019-nCoV) OR ‘COVID-19 Vaccine’ OR (Vaccine AND COVID-19) OR ‘SARS Coronavirus 2 Vaccines’) |
| SCOPUS |
| ALL(("myasthenia gravis" OR ("Myasthenia Gravis" AND Ocular) OR "Ocular Myasthenia Gravis" OR ("Myasthenia Gravis" AND Generalized) OR "Generalized Myasthenia Gravis" OR "Muscle-Specific Receptor Tyrosine Kinase Myasthenia Gravis" OR "Muscle Specific Receptor Tyrosine Kinase Myasthenia Gravis" OR "Muscle-Specific Tyrosine Kinase Antibody Positive Myasthenia Gravis" OR "Muscle Specific Tyrosine Kinase Antibody Positive Myasthenia Gravis" OR "MuSK MG" OR "MuSK Myasthenia Gravis" OR ("Myasthenia Gravis" AND MuSK) OR "Anti-MuSK Myasthenia Gravis" OR "Anti MuSK Myasthenia Gravis" OR ("Myasthenia Gravis" AND Anti-MuSK)))  AND  ALL((“COVID 19 Vaccines” OR (Vaccines AND COVID-19) OR “COVID-19 Virus Vaccines” OR “COVID 19 Virus Vaccines” OR (Vaccines AND “COVID-19 Virus”) OR (“Virus Vaccines” AND COVID-19) OR “COVID-19 Virus Vaccine” OR “COVID 19 Virus Vaccine” OR (Vaccine AND “COVID-19 Virus”) OR (“Virus Vaccine” AND COVID-19) OR “COVID19 Virus Vaccines” OR (Vaccines AND “COVID19 Virus”) OR (“Virus Vaccines” AND COVID19) OR “COVID19 Virus Vaccine” OR (Vaccine AND “COVID19 Virus”) OR (“Virus Vaccine” AND COVID19) OR “COVID19 Vaccines” OR (Vaccines AND COVID19) OR “COVID19 Vaccine” OR (Vaccine AND COVID19) OR “SARS-CoV-2 Vaccines” OR “SARS CoV 2 Vaccines” OR (Vaccines AND SARS-CoV-2) OR “SARS-CoV-2 Vaccine” OR “SARS CoV 2 Vaccine” OR (Vaccine AND SARS-CoV-2) OR “SARS2 Vaccines” OR (Vaccines AND SARS2) OR “SARS2 Vaccine” OR (Vaccine AND SARS2) OR “Coronavirus Disease 2019 Vaccines” OR “Coronavirus Disease 2019 Vaccine” OR “Coronavirus Disease 2019 Virus Vaccine” OR “Coronavirus Disease 2019 Virus Vaccines” OR “Coronavirus Disease-19 Vaccines” OR “Coronavirus Disease 19 Vaccines” OR (Vaccines AND “Coronavirus Disease-19”) OR “Coronavirus Disease-19 Vaccine” OR “Coronavirus Disease 19 Vaccine” OR (Vaccine AND “Coronavirus Disease-19”) OR “COVID 19 Vaccine” OR (Vaccine AND “COVID 19”) OR “2019-nCoV Vaccine” OR “2019 nCoV Vaccine” OR (Vaccine AND 2019-nCoV) OR “2019 Novel Coronavirus Vaccines” OR “2019 Novel Coronavirus Vaccine” OR “2019-nCoV Vaccines” OR “2019 nCoV Vaccines” OR (Vaccines AND 2019-nCoV) OR “COVID-19 Vaccine” OR (Vaccine AND COVID-19) OR “SARS Coronavirus 2 Vaccines”)) |
| WOS |
| TS=((“myasthenia gravis” OR (“Myasthenia Gravis” AND Ocular) OR “Ocular Myasthenia Gravis” OR (“Myasthenia Gravis” AND Generalized) OR “Generalized Myasthenia Gravis” OR “Muscle-Specific Receptor Tyrosine Kinase Myasthenia Gravis” OR “Muscle Specific Receptor Tyrosine Kinase Myasthenia Gravis” OR “Muscle-Specific Tyrosine Kinase Antibody Positive Myasthenia Gravis” OR “Muscle Specific Tyrosine Kinase Antibody Positive Myasthenia Gravis” OR “MuSK MG” OR “MuSK Myasthenia Gravis” OR (“Myasthenia Gravis” AND MuSK) OR “Anti-MuSK Myasthenia Gravis” OR “Anti MuSK Myasthenia Gravis” OR (“Myasthenia Gravis” AND Anti-MuSK)))  AND  TS=((“COVID 19 Vaccines” OR (Vaccines AND COVID-19) OR “COVID-19 Virus Vaccines” OR “COVID 19 Virus Vaccines” OR (Vaccines AND “COVID-19 Virus”) OR (“Virus Vaccines” AND COVID-19) OR “COVID-19 Virus Vaccine” OR “COVID 19 Virus Vaccine” OR (Vaccine AND “COVID-19 Virus”) OR (“Virus Vaccine” AND COVID-19) OR “COVID19 Virus Vaccines” OR (Vaccines AND “COVID19 Virus”) OR (“Virus Vaccines” AND COVID19) OR “COVID19 Virus Vaccine” OR (Vaccine AND “COVID19 Virus”) OR (“Virus Vaccine” AND COVID19) OR “COVID19 Vaccines” OR (Vaccines AND COVID19) OR “COVID19 Vaccine” OR (Vaccine AND COVID19) OR “SARS-CoV-2 Vaccines” OR “SARS CoV 2 Vaccines” OR (Vaccines AND SARS-CoV-2) OR “SARS-CoV-2 Vaccine” OR “SARS CoV 2 Vaccine” OR (Vaccine AND SARS-CoV-2) OR “SARS2 Vaccines” OR (Vaccines AND SARS2) OR “SARS2 Vaccine” OR (Vaccine AND SARS2) OR “Coronavirus Disease 2019 Vaccines” OR “Coronavirus Disease 2019 Vaccine” OR “Coronavirus Disease 2019 Virus Vaccine” OR “Coronavirus Disease 2019 Virus Vaccines” OR “Coronavirus Disease-19 Vaccines” OR “Coronavirus Disease 19 Vaccines” OR (Vaccines AND “Coronavirus Disease-19”) OR “Coronavirus Disease-19 Vaccine” OR “Coronavirus Disease 19 Vaccine” OR (Vaccine AND “Coronavirus Disease-19”) OR “COVID 19 Vaccine” OR (Vaccine AND “COVID 19”) OR “2019-nCoV Vaccine” OR “2019 nCoV Vaccine” OR (Vaccine AND 2019-nCoV) OR “2019 Novel Coronavirus Vaccines” OR “2019 Novel Coronavirus Vaccine” OR “2019-nCoV Vaccines” OR “2019 nCoV Vaccines” OR (Vaccines AND 2019-nCoV) OR “COVID-19 Vaccine” OR (Vaccine AND COVID-19) OR “SARS Coronavirus 2 Vaccines”)) |
